# RANTES Gene G-403A Polymorphism and Coronary Artery Disease: A Meta Analysis of Observational Studies

**DOI:** 10.1371/journal.pone.0047211

**Published:** 2012-10-10

**Authors:** Jun Liu, Yan-Jun Jia, Xiao-Lin Li, Rui-Xa Xu, Cheng-Gang Zhu, Yuan-Lin Guo, Na-Qiong Wu, Jian-Jun Li

**Affiliations:** Division of Dyslipidemia, State Key Laboratory of Cardiovascular Disease, Fu Wai Hospital, National Center for Cardiovascular Diseases, Chinese Academy of Medical Sciences, Peking Union Medical College, Beijing, China; University of Nevada School of Medicine, United States of America

## Abstract

**Objective:**

The G-403A polymorphism in RANTES gene may be involved in the development of coronary artery disease (CAD) through increasing RANTES-mediated leukocyte trafficking and activation. However, studies investigating the relationship between G-403A polymorphism and CAD yielded contradictory and inconclusive results. In order to shed some light on these inconsistent findings, a meta analysis was performed to clarify the role of G-403A polymorphism of RANTES gene in the susceptibility of CAD.

**Methods:**

A systemic literature search of PubMed and EMBASE was conducted from their inception to March 23, 2012, to retrieve related studies. In addition, Conference Proceedings Citation Index-Science was searched, authors of relevant studies were contacted, and reference lists of the included studies and their related citations in PubMed were reviewed for additional pertinent studies.

**Results:**

A total of 8 eligible studies were identified, with a total of 4252 CAD cases and 2150 controls. There was no evidence of significant association between G-403A polymorphism and CAD risk in any genetic model or pairwise comparisons (additive model: OR = 1.046, 95% CI = 0.883–1.239, I^2^ = 65.9%; recessive model: OR = 1.140, 95% CI = 0.774–1.678, I^2^ = 53.1%; dominant model: OR = 1.000, 95% CI = 0.820–1.21), I^2^ = 62.6%; AA vs GG: OR = 1.141, 95% CI = 0.734–1.773, I^2^ = 61.2%; GA vs GG: OR = 0.993, 95% CI = 0.800–1.232, I^2^ = 64.6%). Subgroup analysis and meta regression indicated that ethnicity and genotyping method accounted for the significant heterogeneity among studies. In the stratified analysis by ethnic group, G-403A polymorphism was found to be associated with increased CAD risk in Caucasian population whereas its protective role was observed in Asian population in some but not all comparisons.

**Conclusion:**

Data from the current meta-analysis do not support the existence of a relationship between G-403A polymorphism and the development of CAD, and large sample size study employing unified genotyping method is needed to further evaluate the influence of G-403A polymorphism on susceptibility of CAD.

## Introduction

It has been widely accepted that atherosclerosis is an inflammatory disease mediated by intense immunological activity, a process characterized with the accumulation and activation of the inflammatory and immune cells within the vessel wall [1]. The extravasation of the circulating monocyte and lymphocyte from the peripheral blood to the intima is the most pronounced event in the early stages of atherogenesis [2]. The recruitment of these cells to vascular wall is mainly guided by endothelial leukocyte adhesion molecules and chemoattractants [3]. Given their fundamental role in the inception of atherosclerosis, chemokines and their receptors have been the focus of intensive researching during the past decades.

Regulated on activation, normal T cell expressed and secreted (RANTES), also known as CCL5, belongs to the CC chemokine family and has strong chemoattractant and activating properties for T-lymphocytes and macrophages [4]. Several lines of evidence have indicated that RANTES involved in the formation [5], exacerbation [6] and instability of atherosclerotic lesions [7]. Recently, a polymorphism in the RANTES promoter region (G-403A) has been shown to increase the promoter activity, result in increased expression of RANTES and affect the RANTES-mediated inflammatory disease [8], including atherosclerosis. Some clinic-based observational studies have reported that G-403A polymorphism was associated with the susceptibility of coronary artery disease (CAD) [9], [10]. However, an observational study in Korea have inconsistently shown a decreased risk of CAD in individuals with G-403A polymorphism [11]. Still other studies could not draw any firm conclusion about the existence of association between G-403A polymorphism and CAD because of the limitation in sample size [12]–[14]. We therefore performed a meta analysis of available studies with a case-control or cohort design to clarify the role of the G-403A polymorphism in RANTES gene in development of CAD.

**Table 1:** Description of search strategy for electronic databases.

PubMed
(“RANTES” [All Fields] OR CCL5 [All Fields] OR “CC chemokine ligand-5” [All Fields] OR “chemokine CCL5” [MeSH Terms]) AND (“coronary artery disease” [All Fields] OR “acute coronary syndromes” [All Fields] OR “myocardial infarction” [All Fields] OR “coronary atherosclerosis” [All Fields] OR “coronary artery disease” [MeSH Terms])
EMBASE
('rantes'/exp OR 'cc chemokine ligand 5': ab OR 'ccl5': ab) AND ('coronary artery disease'/exp OR 'acute heart infarction'/exp OR 'acute coronary syndrome'/exp OR 'coronary artery disease': ab OR 'myocardial infarction': ab OR 'acute coronary syndrome': ab)

## Methods

### Search Strategy and Selection Criteria

PubMed, and EMBASE (from their inception to March 23, 2012) were searched to identify potentially relevant studies. A search strategy combining both the Medical Subject Heading and text words was used. No language restriction was applied and non–English-language articles were translated before further analysis. The search strategy is listed in table 1. Reference lists of all the included articles, the related articles of each included study generated by PubMed and relevant reviews were also screened to avoid the omitting of any potentially relevant studies. Authors of abstracts were contacted to obtain missing data when necessary. In addition, conference proceedings were searched in Conference Proceedings Citation Index-Science (CPCI-S) and authors of relevant studies were contacted via email and asked for any other published or unpublished studies that might contribute to the meta-analysis.

**Figure 1:**
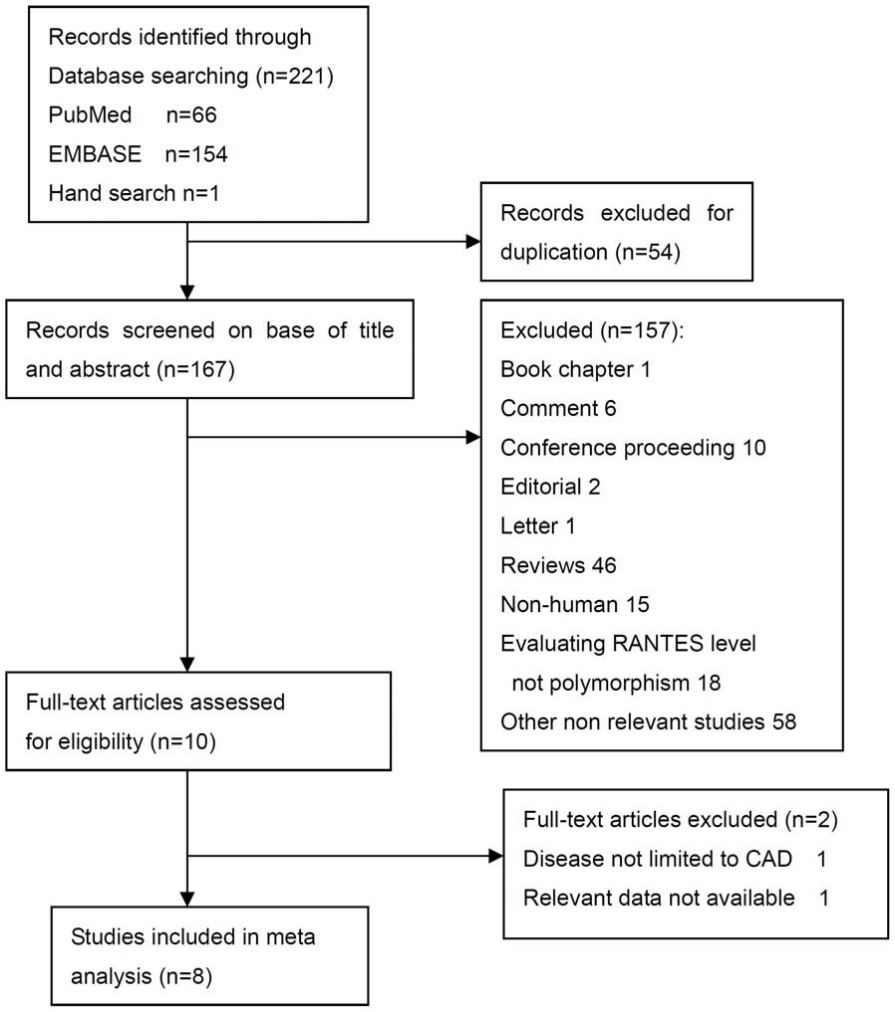
Flow diagram of literature identification and filtration in the systematic review.

Studies were included for the meta-analysis if they satisfied the following criteria: 1) they evaluate the association between G-403A polymorphism in RANTES gene and CAD; 2) they included individuals with CAD and those without CAD; 3) the baseline characteristics were comparable between the two distinct population; and 4) the size of sample, source of control, distribution of alleles, and frequency of genotype were all reported or can be calculated using the data provided in studies.

**Table 2:** Characteristics of the studies included in meta-analysis.

			Mean Age, y		Gender§	Definition of	Control	Genotyping	Subjects n		Quality
Author	Year	Ethnicity	Cases	Controls	Component	Cases	Source	Method	Cases	Controls	Score
Berg^12^	2009	Caucasians	60	57	106/24/36/64	CAD(≥50% stenosis of ≥1 major vessel	Hospitalbased	SSCP	130	100	7
Jang^11^	2007	Asians	54.8	53.8	NA	CAD(≥50% stenosis of ≥1major vessel), previous MI	Populationbased	TaqMan	553	416	9
Park^16^	2007	Asians	62.2	62.6	129/41/114/56	AMI	Populationbased	SNaPshot	170	170	9
Simeoni^9^	2004	Caucasians	63.8	56.9	NA	CAD(≥50% stenosis of ≥1 major vessel	Hospitalbased	RFLP	2231	530	8
Szalai^17^	2001	Caucasians	57.6	58.9	242/76/240/80	CAD(≥70% stenosis of ≥2 Major vessels	Populationbased	RFLP	318	320	8
Tavakkoly-Bazzaz^13^	2011	Caucasians	63	55	115/76/88/40	CAD(≥50% stenosis in all 3 major vessel	Hospitalbased	RFLP	191	128	7
Tereshchenko^14^	2011	Caucasians	55.8	40.3	353/114/174/163	MI	Populationbased	PCR-SSP	467	337	8
Vogiatzi^10^	2009	Caucasians	64	62	171/21/106/43	previous CAD	Populationbased	RFLP	192	149	8

NA, data not available; CAD, coronary artery disease; AMI, acute myocardial infarction; MI, myocardial infarction; SSCP, single-strand conformation polymorphism; RFLP, restriction fragment length polymorphisms; PCR-SSP, polymerase chain reaction with sequence-specific primers.

§ Gender component: number of male cases/number of female cases/number of male controls/number of female controls.

**Table 3:** The distribution of RANTES genotype among cases and controls included in meta-analysis.

	Genotypes for Cases			Genotypes for Controls			Case		Control		Freq. of A Allele		Test for HWE	
Author	GG	GA	AA	GG	GA	AA	A	G	A	G	Cases	Controls	?^2^	P
Berg	91	NA	NA	66	NA	NA	NA	NA	NA	NA	NA	NA	NA	NA
Jang	229	242	82	135	211	70	406	700	351	481	0.37	0.42	0.659	0.4168
Park	82	70	17	71	67	24	104	234	115	209	0.31	0.35	1.519	0.2178
Simeoni	1439	706	86	370	137	23	878	3584	183	877	0.20	0.17	4.799	0.0285
Szalai	199	106	13	221	93	6	132	504	105	535	0.21	0.16	1.135	0.2868
Tavakkoly-Bazzaz	116	61	14	74	49	5	89	293	59	197	0.23	0.23	0.804	0.3700
Tereshchenko	310	135	22	224	101	12	179	755	125	549	0.19	0.19	0.022	0.8828
Vogiatzi	96	83	13	83	65	1	109	275	67	231	0.28	0.22	9.427	0.0021

NA, data not available; CAD, coronary artery disease; HWE, Hardy-Weinberg equilibrium.

### Data Extraction and Quality Assessment

Two investigators independently extracted data from each study with a predefined review form and discrepancies were resolved by consensus of all investigators. Information extracted included: author, year of publication, study design, country of origin, gender composition, mean age, diagnosis of CAD, selection of control (population-based or hospital-based), sample size, ethnicity, and genotyping method.

**Table 4:** Summary odds ratio (95%CI) and I^2^ for G-403A polymorphism and coronary artery disease under additive, recessive and dominant model.

	Additive model		Recessive model		Dominant model	
Subgroup	N§	OR (95% CI) I^2^(%)	N	OR (95% CI) I^2^(%)	N	OR (95% CI) I^2^(%)
Source of controls						
Population based	5	1.026 (0.815–1.291) 72.5	5	1.214 (0.697–2.113) 63.6	5	0.978 (0.748–1.279) 67.7
Hospital based	2	1.144 (0.975–1.341) 0	2	1.136 (0.552–2.337) 45.2	3	1.064 (0.795–1.423) 42.7
Ethnicity						
Caucasians	5	1.173 (1.045–1.316) 0	5	1.582 (0.887–2.822) 53.7	6	1.155 (1.007–1.324) 4.7
Asians	2	0.798 (0.680–0.936) 0	2	0.808 (0.594–1.100) 0	2	0.717 (0.572–0.900) 0
Cases definition						
CA	4	1.053 (0.822–1.348) 76.8	4	1.078 (0.728–1.596) 41.0	5	0.985 (0.728–1.333) 76.3
Non-CA	3	1.041 (0.794–1.364) 57.1	3	1.434 (0.484–4.255) 74.6	3	1.013 (0.819–1.253) 0
HWE violation						
Yes	2	1.210 (1.034–1.416) 0	2	2.544 (0.207–31.323) 82.9		NA
No	5	0.972 (0.796–1.187) 62.5	5	1.093 (0.726–1.647) 44.3		NA
Genotyping method						
RFLP	4	1.209 (1.063–1.376) 0	4	1.850 (0.803–4.262) 65.4	4	1.233 (1.059–1.435) 0
Non-RFLP	3	0.869 (0.730–1.034) 34.1	3	0.876 (0.644–1.191) 8.6	4	0.818 (0.672–0.996) 18.8
Overall	7	1.046 (0.883–1.239) 65.9	7	1.140 (0.774–1.678) 53.1	8	1.000 (0.820–1.219) 62.6

NA,data not available; CA, coronary angiography; HWE, Hardy-Weinberg equilibrium;

RFLP, restriction fragment length polymorphisms.

§ N:The number of studies included in subgroup.

**Table 5:** Summary OR (95%CI) and I^2^ for G-403A polymorphism and coronary artery disease under AA vs GG and GA vs GG contrasts.

Subgroup	AA vs GG		GA vs GG	
	N§	OR (95% CI) I^2^(%)	N	OR (95% CI) I^2^(%)
Source of controls				
Population based	5	1.208 (0.631–2.314) 71.3	5	0.949 (0.749–1.201) 54.4
Hospital based	2	1.084 (0.671–1.754) 8.4	2	1.074 (0.655–1.759) 72.9
Ethnicity				
Caucasians	5	1.595 (0.918–2.772) 48.8	5	1.129 (0.948–1.344) 27.8
Asians	2	0.672 (0.480–0.940) 0	2	0.737 (0.568–0.955) 10.2
Cases definition				
CA	5	1.088 (0.661–1.792) 59.1	4	0.987 (0.687–1.418) 81.7
Non-CA	3	1.442 (0.464–4.480) 75.4	3	1.104 (0.713–1.710) 0
HWE violation				
Yes	2	2.695 (0.232–31.370) 82.0	2	1.279 (1.054–1.552) 0
No	5	1.054 (0.643–1.727) 57.9	5	0.900 (0.714–1.136) 51.9
Genotyping method				
RFLP	4	1.879 (0.844–4.181) 61.8	4	1.183 (0.975–1.435) 23.6
Non-RFLP	3	0.780 (0.525–1.158) 30.2	3	0.821 (0.645–1.045) 34.2
Overall	7	1.141 (0.734–1.773) 61.2	7	0.993 (0.800–1.232) 64.6

Abbreviations as in Table 4.

§ N:The number of studies included in subgroup.

**Figure 2:**
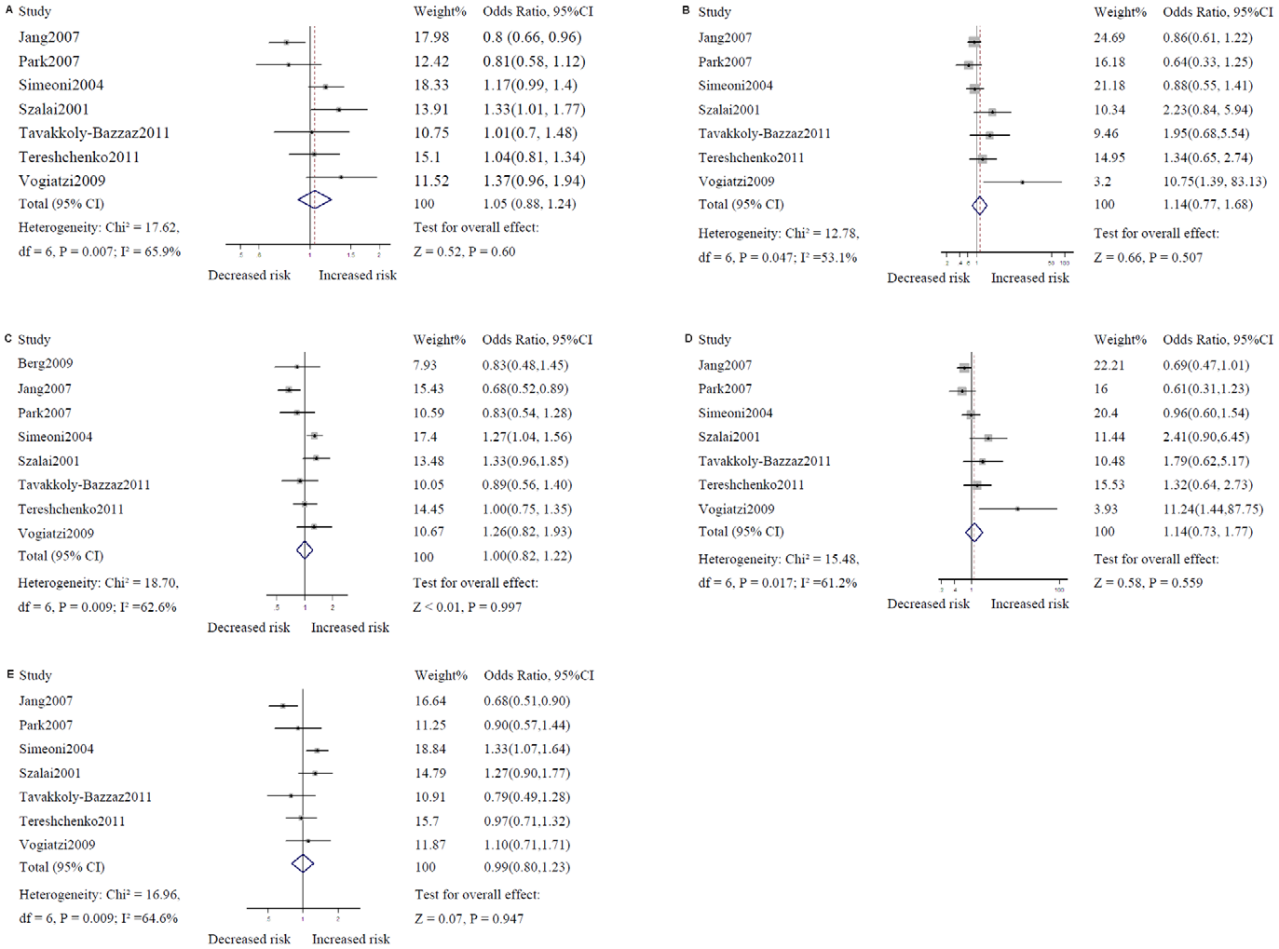
Results of association between the G-403A polymorphism and coronary artery disease. The overall odds ratio (OR) was estimated with DerSimonian-Laird random effects model. The ORs of individual studies are shown as squares, with the size proportional to the weight of each study in the overall estimate; 95% confidence intervals (CIs) are shown as horizontal lines. The pooled OR and its 95% CI are shown as a dashed vertical line and a diamond, respectively. A, Additive model. B, Recessive model. C, Dominant model. D, AA versus GG. E, GA versus GG.

The quality of each study was assessed independently by two investigators using the Newcastle-Ottawa quality assessment scale [15]. The quality of case control studies were evaluated the in the following three major components: selection of case and controls, comparability of cases and controls, and ascertainment of exposure. For cohort studies, the items assessed included selection of cohorts, comparability of cohorts, and assessment of outcome.

**Figure 3:**
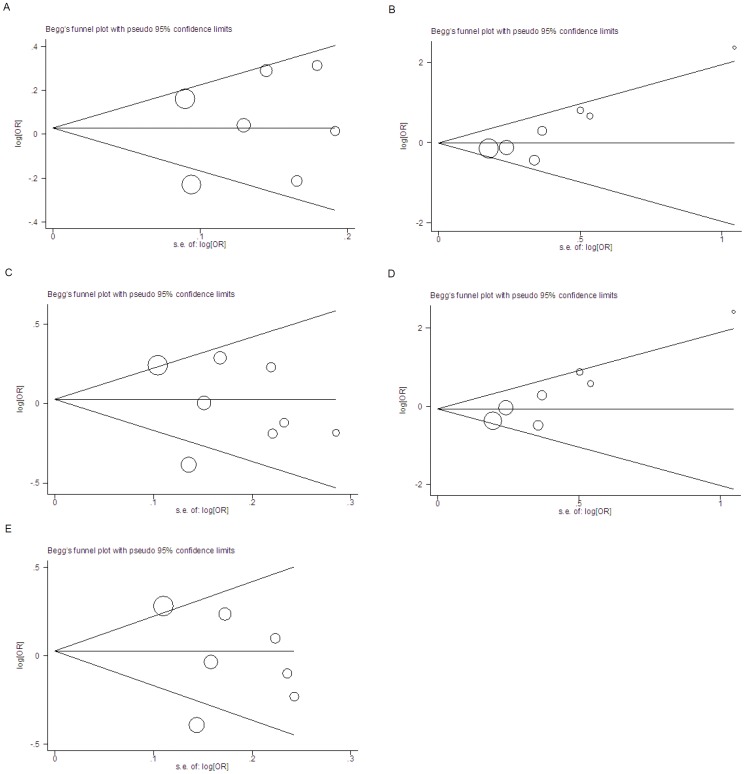
Begg’s funnel plot of publication bias with pseudo 95% confidence limits. The horizontal line in the funnel plot indicates the random effects summary estimate, while the sloping lines indicate the expected 95% confidence intervals for a given standard error, assuming no heterogeneity between studies. Each study is represented by a circle, the area of which represents the study’s precision. A, Additive model (Egger’s test P = 0.643). B, Recessive model (Egger’s test P = 0.020 ). C, Dominant model (Egger’s test P = 0.519). D, AA versus GG (Egger’s test P = 0.014). E, GA versus GG (Egger’s test P = 0.429).

### Statistical Analysis

Deviation from Hardy-Weinberg equilibrium (HWE) in the control group was examined using a two tailed chi-square test. Association between G-403A polymorphism and CAD were tested under the following three genetic models: additive, recessive, and dominant. In addition, contrast of genotype AA versus GG, and GA versus GG were also evaluated.

**Table 6:** Univariable meta regression analysis for heterogeneity under the additive model.

Variable	Coefficient	P-Value	τ	I^2^(% res)	Adj R^2^(%)
Source of controls (0 = population based, 1 = hospital based)	−0.08346	0.690	0.0363	66.71	−20.03
Ethnicity (0 = Caucasians, 1 = Asians)	−0.38508	0.012	0	0	100
Cases definition (0 = Non CA, 1 = CA)	0.00967	0.961	0.03925	71.61	−29.78
HWE violation (0 = yes, 1 = no)	−0.24653	0.204	0.02301	55.56	23.91
Genotyping method (0 = Non-RFLP, 1 = RFLP)	0.33917	0.019	0.00094	0	96.89

CA, coronary angiography; HWE, Hardy-Weinberg equilibrium; RFLP, restriction fragment length polymorphisms.

The statistical heterogeneity among studies was assessed with Q statistic, and I^2^ test was used to quantify inconsistency. A Q statistic P value <0.10 or an I^2^ value≥50% was considered to represent significant statistical heterogeneity. Data were combined using a Mantel-Haenszel fixed effect method in the absence of heterogeneity; otherwise, DerSimonian and Laird random effect method was used.

A sensitivity analysis was conducted, after excluding each of the studies in turn, to evaluate whether the results could have been affected significantly by a single study. To explore the potential source of heterogeneity among individual studies and test the effects of study characteristics and methodological quality on the overall effect estimates, subgroup analyses and meta regression were performed stratified by the following factors: source of controls (population or hospital based), ethnicity (Caucasians or Asians), definition of cases (coronary angiography (CA) or non-CA), violation of HWE (yes or no), and genotyping methods.

Potential publication bias was examined by the asymmetry linear regression of Egger's test and displayed as funnel plot of precision (standard error of log RR) against the treatment effect (displayed as RR on a logarithmic scale). Statistical analyses were undertaken using Stata version 12.0 (StataCorp, College Station, TX).

## Results

### Literature Review and Description of Included Studies

A total of 220 potentially relevant studies were identified from the following databases in our initial literature search: 66 from PubMed, 154 from EMBASE. Our hand search of the relevant reviews identified an additional citation. Our retrieving in CPCI-S and contacting with authors identified no additional studies. After screening the titles or abstracts, 211 studies were excluded because of duplicate data, other types of publications (book chapter, comment, conference proceeding, editorial, letter, or review), or non-relevance research (non-human study or evaluating RANTES level not polymorphism) (figure 1). The full-length papers of the remaining 10 studies were retrieved and assessed for eligibility. Of the retrieved studies, a total of eight [9]–[14], [16], [17] met our inclusion criteria and were included in our systematic review. The remaining 2 studies were excluded because the disease was not limited to CAD [18], or because the frequency of genotype was not available [19].

All studies included in the present meta analysis used a case-control design, with a total of 4252 CAD cases and 2150 controls. Characteristics of the included studies are summarized in table 2. Six studies were conducted in Caucasian population, while two were conducted in Asian population. Diverse genotyping methods were used, including single-strand conformation polymorphism (SSCP), TaqMan, SNaPshot, restriction fragment length polymorphisms (RFLP), and polymerase chain reaction with sequence-specific primers (PCR-SSP). The definition of cases varied among studies, but most employed coronary angiography for diagnosis of CAD [9], [11]–[13], [17]. Five studies recruited population-based controls and three [9], [12], [13] recruited patients without CAD as controls. The distribution of RANTES genotype and A allele frequencies among CAD cases and controls are given in table 3. Seven of the eight studies reported data enabling formal testing of whether genotype frequencies in the control group deviated from HWE. In two studies, the genotype distributions in control groups were not consistent with that expected under HWE.

Using the quality assessment instrument, case-control study was assigned a maximum of 4 points for selection of case and controls, a maximum of 2 points for comparability of cases and controls, and a maximum of 3 points for ascertainment of exposure. Consequently, the theoretical maximum sum score of 9 points could be obtained if a study fulfilled all of the aforementioned criteria in quality assessment. Based on the quality assessment scale for case control studies, two studies had the maximum score of 9 points, four scored 8 points, and two scored 7 points, with a mean score of 8 (table 2).

### Data Synthesis and Subgroup Analyses

A DerSimonian and Laird random effect method was employed in the estimation of overall odds ratio (OR) because of the significant heterogeneity in our initial data synthesis with fixed effect model. Table 4 and table 5 presents the results of meta analysis for the association of G-403A polymorphism and the susceptibility of CAD under different genetic models and contrasts. When all studies were included in the quantitative analysis, the results did not support the existence of relation between G-403A polymorphism and CAD. The overall ORs for the additive, recessive, as well as dominant genetic model, AA versus GG, and GA versus GG were 1.046 (95%CI: 0.883–1.239), 1.140 (95%CI: 0.774–1.678), 1.000 (95%CI: 0.820–1.219), 1.141 (95%CI: 0.734–1.773), 0.993 (95%CI: 0.800–1.232), respectively (figure 2). In the subgroup analysis under additive model, G-403A polymorphism was found to be correlated with increased risk of CAD when the estimation of pooled OR was restricted to Caucasians ethnic group (OR = 1.173, 95%CI: 1.045–1.316), studies violating HWE (OR = 1.210, 95%CI: 1.034–1.416), or studies using RFLP as genotyping method (OR = 1.209, 95%CI: 1.063–1.376). However, the G-403A polymorphism was found to be associated with decreased risk of CAD in Asians ethnic group (OR = 0.798, 95%CI: 0.680–0.936). In the subgroup analysis under dominant model, statistically significant but contradictory results were found among Caucasians ethnic group (OR = 1.155, 95%CI: 1.007–1.324), Asians ethnic group (OR = 0.717, 95%CI: 0.572–0.900), studies using RFLP genotyping method (OR = 1.233, 95%CI: 1.059–1.435), and studies using Non-RFLP genotyping method (OR = 0.818, 95%CI: 0.672–0.996). The decreased risk of CAD was also found in the Asians ethnic group under both AA vs GG and GA vs GG contrasts (OR = 0.672, 95% CI: 0.480–0.940; OR = 0.737, 95% CI: 0.568–0.955, respectively). Besides, combined data from studies violating HWE suggested a positive association of G-403A polymorphism with risk of CAD under GA vs GG contrast (OR = 1.279, 95% CI: 1.054–1.552). The remaining pooled ORs from other subgroup analysis did not reach statistical significance.

### Sensitivity and Publication Bias

The results of meta analysis remained non-significant when each study was sequentially omitted from the analysis, indicating that the results of the present meta analysis were stable. The shape of funnel plots was symmetrical in the additive and dominant genetic model, as well as in contrast of GA versus GG, and the Egger’s test P value was 0.643, 0.519, and 0.429 respectively, suggesting no evidence of publication bias (Figure 3). However, obvious funnel plot asymmetry was found in recessive genetic model and the contrast of AA versus GG, with a significant Egger’s test P value (P = 0.020, P = 0.014 respectively), implying the existence of publication bias.

### Meta Regression

Since there was significant heterogeneity among individual studies, a univariable regression was conducted to explore the predefined possible source of heterogeneity under additive model. The results of univariable regression suggested that ethnicity and genotyping method were the significant source of heterogeneity (P = 0.012, 0.019, respectively), while the heterogeneity in the overall ORs could not be accounted for by source of controls, difference in cases definition or deviation from HWE (table 6). Including ethnicity and genotyping method in a multivariable regression model showed that those two variables can explain all of the heterogeneity (F = 7.86, P = 0.0411, τ = 0, I^2^-residual = 0%, Adj R^2^ = 100%).

## Discussion

The present meta-analysis, which involved 4252 CAD cases and 2150 controls, showed no evidence of an association between the G-403A polymorphism and CAD. Of the five overall ORs estimated under different genetic models and genotype contrasts, three showed a trend toward increased risk for CAD (OR>1), one showed an opposite trend toward decreased risk for CAD (OR<1), and the remaining one was a null value (OR = 1). Therefore it was not possible to draw any firm conclutions from these contradictory and not statistically significant results.

Significant heterogeneity existed among studies in all five comparisons, and subgroup analysis as well as meta regression confirmed that ethnicity and genotyping method variation account for all of the heterogeneity. Besides, subgroup analysis stratified by ethnicity, genotyping method, and departure from HWE provided significant and contradictory results in some, but not all, comparisons. G-403A polymorphism seemed to be a risk factor for the development of CAD in Caucasians ethnic group (under additive and dominant model), whereas its protective role in CAD was shown in Asians ethnic group (in all comparisons except recessive model), indicating that the role of G-403A polymorphism in the development of CAD may be ethnicity dependent. However, given the limited studies in Asians ethnic group, such implication needs further scientific justification based on large sample. Similarly, opposite results were also obtained among studies employing different genotyping method. In studies using RFLP method, G-403A polymorphism was shown to be a risk factor (under additive and dominant model) while in studies using non-RFLP method, it was shown to be protective for the development of CAD (under dominant model). Although no heterogeneity (I^2^ = 0% for RFLP subgroup under both additive and dominant model) or only minor heterogeneity (I^2^ = 18.8% for no-RFLP subgroup under dominant model) was presented in these statistically significant results, due to the variation in sensitivity and accuracy of different genotyping method, choosing an appropriate one is critical in SNP analysis. RFLP was the most often used method for genotyping RATENT in the present meta analysis because of its relative simplicity. However, RFLP is not a direct method and its discriminatory power depends on the restriction enzymes’ success in achieving recognition and cleavage at restriction sites. Besides, the high reaction temperature needed for RFLP might decrease the sensitivity of assay. Other genotyping methods, such as SSCP, SNaPshot, Taqman and PCR-SSP, were also employed in the studies included in the present meta analysis. Although SSCP has been widely used for genotyping, it is only suitable for screening sequence variations because of its relative low sensitivity [20] and inability to localize those variations to particular nucleotides. It has been demonstrated that SNaPshot [21], Taqman [22] and PCR-SSP [20] could provide high sensitivity and accuracy in SNP genotyping under optimized conditions. Hence, SNaPshot, Taqman and PCR-SSP are more reliable and accurate genotyping method for SNP detection compared with RFLP and SSCP. To minimize assay variation and increase statistical power, further studies with identical and reliable genotyping method and large sample will be required to confirm the association between G-403A polymorphism and CAD risk. Under additive model and GA vs GG comparison, pooled ORs for studies departing from HWE achieved statistical significance and indicated an increased risk of CAD for individuals with G-403A polymorphism. Because departure from HWE could be attributed to some potential bias such as genotyping error, possible ethnic admixture in the population or selection bias in choosing subjects, these results may not valid and must be interpreted with caution.

The G-403A polymorphism is caused by the substitution of adenine for guanine in the promoter region of the RANTES gene. There has been evidence that the A-403 mutant can lead to increased transcription of RANTES [8] and was associated with high levels of serum RANTES [11]. It has been proven that RANTES was delivered by activated platelets or platelet microparticles in initiation of the atherogenetic process [23], [24]. Subsequently, RANTES released by platelets deposited on the surface of inflamed endothelium, promoting circulating monocytes recruitment onto endothelial cells [23]. It is therefore reasonable to hypothesise that G-403A polymorphism is associated with the susceptibility of CAD. However, the present meta analysis suggested that there was a lack of association between G-403A polymorphism and the development of CAD. Given the complexity of the etiology of CAD and the interplay between genetic background and exposures to environmental factors, the following factors may contribute to the lack of an association between the G-403A polymorphism and susceptibility of CAD. First, the G-403A polymorphism is not the only mutants found in the promoter region of RANTES gene and another mutant, C-28G polymorphism, has also been found to exist and affect its transcription activity [25]. Hence, merely considering the role of G-403A polymorphism without excluding the influence of C-28G polymorphism may mask the possible relationship between the former and CAD. Second, RANTES exerts its chemotactic function by binding to several receptors, including CCR1, CCR3, as well as CCR5 [26] and other genes regulating the expression of these receptors may influence the association between G-403A polymorphism and susceptibility of CAD. Third, since CAD is a complex and multifactorial disease, the effect of single genetic factor on the risk of CAD may be more pronounced in the presence of conventional cardiovascular risk factors such as hypertension, diabetes, and hypercholesterolemia. Although these risk factors were all well comparable within each single study, it was not excluded that the variability in baseline characteristics may exist among studies and any imbalance in these risk factors would distort the association of G-403A polymorphism with CAD.

There are some limitations with this meta-analysis that should be considered when interpreting the clinical significance of the results. A notable limitation is that all analyses were conducted at the study level because of the unavailability of individual patient data from the original studies. Therefore, our analysis was based on unadjusted estimates and classic CAD risk factors, including smoking, hypertension, diabetes, and dyslipidemia, can not be adjusted to provide more precise overall risk estimates. Besides, environmental factors such as lifestyle, diet, alcohol consumption, and stress were also unavailable and limited the evaluation of gene-environment interactions. Additionally, unpublished studies were not included in this meta analysis and both the asymmetric funnel plot and Egger’s test indicated the existence of publication bias in two of the five comparisons. Finally, it was likely that the statistically significant results observed in certain subgroup might be due to chance or confounding because of the limited studies included in that subgroup.

In conclusion, there was no evidence in support of the hypothesis that the G-403A polymorphism had any influence on the susceptibility of CAD in the current meta-analysis. Although subgroup analysis and meta regression suggested possible different and statistically significant influence of this mutant on CAD in Caucasian and Asian population, further analysis with more homogeneous sample and unified genotyping method is warranted to clarify this potential impact.
